# MALDI imaging mass spectrometry differentiates basal cell carcinoma from trichoblastoma and trichoepithelioma: A proof of principle study

**DOI:** 10.1371/journal.pone.0323475

**Published:** 2025-05-12

**Authors:** Jennifer M. C. Ranes, Jessica L. Moore, Nathan H. Patterson, Sarah P. Nicholson, Sara Kantrow, Jason Robbins, Richard M. Caprioloi, Jeremy L. Norris, Rami N. Al-Rohil

**Affiliations:** 1 St. George’s University School of Medicine, True Blue, Grenada; 2 Frontier Diagnostics, LLC, Nashville, Tennessee, United States of America; 3 Mass Spectrometry Research Center, Department of Biochemistry, Vanderbilt University, Nashville, Tennessee, United States of America; 4 Pathology Associates of Saint Thomas, Nashville, Tennessee, United States of America; 5 Duke University Department of Pathology and Dermatology, Durham, North Carolina, United States of America; 6 The University of North Carolina Chapel Hill, Chapel Hill, North Carolina, United States of America; Laurentian University, CANADA

## Abstract

**Background:**

Basal cell carcinoma (BCC) comprises a large portion of dermatopathology specimens; however, benign mimics such as trichoblastoma/trichoepithelioma (TB/TE) place accurate diagnosis at risk and consequently lead to inappropriate clinical management and overuse of healthcare resources. This study aims to address the challenges of traditional histopathological evaluation by utilizing matrix-assisted laser desorption ionization imaging mass spectrometry (MALDI IMS).

**Methods and Findings:**

Formalin-fixed paraffin-embedded BCC and TB/TE tissue blocks were taken from archival tissue. A cohort of 69 BCC and TB/TE specimens were identified, each having three concordant diagnoses given by Dermatopathologists after a blinded analysis. H&E stained sections of each specimen were imaged for pathological analysis and uploaded to a digital annotation software with the following classifications: BCC, TB, TE, BCC stroma, TB stroma, and TE stroma. Mass spectra were collected from unstained serial sections guided by the areas annotated by the Dermatopathologists on the H&E stained serial sections. Before informatics, the data from the cohort were divided randomly into a training set (n = 55) and a validation set (n = 14). Prediction models were developed using a support vector machine (SVM) classification model from the training set data.

The platform predicted BCC and TB/TE in model 2 (tumor nests alone) with a sensitivity of 98.9% (95% CI 98.3–99.4%) and specificity of 88.4% (95% CI 78.4–94.5%) at the spectral level in the validation set. Model 1 (stroma alone) had a sensitivity of 46.1% (95% CI 43.0–49.1%) and specificity of 99.2% (95% CI 97.1–99.9%). A combined model 3 (tumor nests and stroma) had a sensitivity of 90.26% (95% CI 89.1%-91.3%) and a specificity of 97.1% (95% CI 94.6% to 98.7%). The limitations of this study included a small sample set, which included easily identifiable cases obtained from a single tissue source.

**Conclusions:**

Our study proves that BCC and TB/TE exhibit different proteomic profiles that one can use to enable accurate differential diagnosis. While our findings are not yet validated for clinical use, this merits further research to support IMS as an ancillary diagnostic tool for adequately and efficiently identifying the most common cutaneous malignancy in the United States. We recommend that future studies obtain a more extensive set of histologically challenging cases from multiple institutions and adequate clinical follow-up to confirm diagnostic accuracy.

## Introduction

As the incidence of Basal cell carcinoma (BCC) rises by 4%-8% annually, it remains steadfast as the most common malignancy in the United States, accounting for up to 30% of all malignancies.[1,2] While patients with Fitzpatrick skin types I and II, light-colored eyes, freckles, blonde or red hair, northern European ancestry, and advanced age are most commonly affected, UV radiation exposure plays a crucial role in developing BCC.[3,4] As age increases, so does the incidence of BCC, with 65 and 67 years being the average age of diagnosis for women and men, respectively.[5] BCC incidence is more common in men than women, demonstrating a 1.5–2:1 ratio.[2] However, recent trends show that an increasing incidence among Americans younger than 40 is particularly prevalent in women.[6]

Additionally, immunosuppression amongst HIV-positive and organ transplant patients confers a 2-time and 50 times greater incidence of BCC, respectively.[7] Genetic syndromes, including Basal cell nevus syndrome (BCNS), Xeroderma pigmentosum, and Bazex-Dupre-Christol syndrome, share characteristic findings of BCC due to the loss of hedge-hog signaling proteins, defects in nucleotide excision repair proteins, and depletion of tumor suppressors, accounting for its increased incidence as well. [8–10]

BCC occurrence in the general population is a result of constitutive activation of the hedgehog intracellular signaling pathway, commonly associated with inactivating mutations of patched homolog 1 (PTCH1) and activating mutations of smoothened homolog (SMOm), followed by UV-specific defects in the p53 tumor suppressor gene and loss-of-function mutations in suppressor of fused homolog (SUFU). [1,11,12]

**Fig 1:**
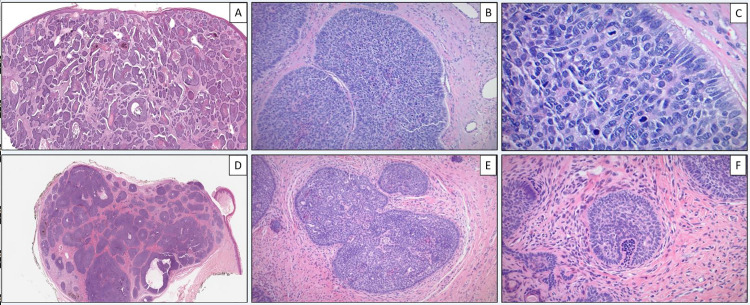
Basal cell carcinoma with a nodular growth pattern showing nested growth pattern of basaloid nests within the dermis and no epidermal connection (A, H&E 20X), the nests grow in an expansile fashion in the dermis and induce some fibrotic changes in the background stroma (B, H&E 100X). On closer inspection, the basaloid nests show peripheral palisading, with apoptotic bodies and mitotic figures, along with some deposition of myxoid substance (C, H&E 200X). An example of trichoblastoma showing the basaloid nests within the dermis with no overlying epidermal connection (D, H&E 20X), Similar to basal cell carcinoma, trichoblastoma also grows in the form of expansile basaloid nests in the dermis with peripheral palisading; however, the difference is mostly subtle histologically represented by a spindled stroma with clefting within the stroma and minimal mitotic activity (E, H&E 100X). The presence of papillary mesenchymal bodies is a helpful feature in trichoblastoma/trichoepithelioma (F, H&E 200X).

BCCs is based on the clinical and pathological risk factors for recurrence established by the National Comprehensive Cancer Network (NCCN) guidelines.[13] These risk factors include location/size, borders, primary vs. recurrent, immunosuppression, site of prior radiotherapy, pathology subtype, and perineural involvement.[14] BCC is classified into lower- and higher-risk histological subtypes, each presenting with a unique clinical appearance, treatment course, and recurrence rate. This study focuses on the most common subtype, nodular BCC, which Trichoblastoma and Trichoepothelioma (TB/TE) often mimic. Comprising 60–80% of all BCCs, nodular BCC appears as pearly or translucent papules or nodules with rolled borders and telangiectasias, most commonly found on the face and neck.[3,15,16] Classically, nodular BCC (Figs 1A–1C) presents as large tumor nests comprised of basophilic-staining proliferating cells with peripheral palisading.[3] Mitoses, apoptosis, intra and peritumoral mucin, clefting, and stromal retraction away from the tumor nodules are also characteristic.[17]

Often mimicking BCC in clinical and histological appearance, TB/TE are benign hair-germ tumors commonly found in adults aged 40 or older.[18] Outside of the general population, individuals with Brooke-Spiegler syndrome and multiple familial trichoepitheliomas (MFT) are predominantly predisposed to these lesions due to mutations affecting tumor suppressor genes, resulting in elevated NFkB signaling.[18]

TB/TE presents similarly to nodular BCC as skin-colored papules or nodules, commonly appearing on sun-exposed areas of the head, trunk, and extremities.[18] Histologically, TB/TE (Figs 1D–1F) exhibits peripheral palisading and spindled fibroblast follicular stroma without mucin with the formation of papillary mesenchymal bodies; however, some variations are present.[18] Typically, TB appears as a well-circumscribed dermal tumor of primitive basaloid cells arranged in nodules, which can vary in size.[18] TE differs in its connection to the epidermis, the mature appearance of tumor cells, and the possible presence of horn cysts.[18] TB/TE generally lacks tumor necrosis, stromal mucin, cytologic atypia, and peripheral clefting; however, TB/TE commonly presents with intrastromal clefting.[19]

While surgical excision is the standard therapy for BCC, Mohs micrographic surgery, electrodesiccation and curettage, cryosurgery, topical imiquimod, topical fluorouracil, photodynamic therapy, radiation therapy, and intralesional treatments are all viable options dependent on the extent of disease, recurrence risk, and patient preference.[3] Resection of BCC with safe margins is ideal given its potential for local aggressive growth; however, the ability to remove benign lesions of TB/TE with shave excision exemplifies the multifaceted importance of accurate diagnostics.[20,21]

The identification of BCC lesions is pertinent to avoiding the over- or under-treatment of these malignancies.[14] Each BCC treatment modality is accompanied by an avoidable cost if properly identified as TB/TE and thus alternatively managed by clinical monitoring of the lesion. Additionally, given TB/TE and BCC are both commonly found in regions of the face, misdiagnosis can result in surgical excision of a benign lesion with the risk of potential unnecessary scarring, adversely affecting the patient both physically and psychologically.

Dermoscopy often falls short in differentiating TB/TE from BCC due to their shared findings of arborizing vessels and gray ovoid nests and globules, thus necessitating histopathologic evaluation.[18] Immunohistochemical evaluation utilizing keratin 20 (CK20) and androgen receptor (AR) staining can help distinguish TB/TE from BCC.[18,22] CK20 stains Merkel cells in TB/TE, while AR positivity suggests BCC. However, this is only sometimes the case as some BCCs demonstrate focal or completely absent AR positivity.[18] Multiple variants of TB also stain positively for CD34 (stromal), BCL-2, and PHLDA1, aiding in its identification; however, overlap remains.[18] An uncommon BCC variant, Fibroepithelioma of Pinkus (FeP), often stains positive for the frequent TB/TE markers CK20 and BCL-2, in addition to the BCC marker AR, further convoluting the immunohistochemical identification of these lesions.[18] The diminished accuracy provided by immunohistochemical markers in the differentiation between these benign and malignant skin lesions, and thus appropriate treatment implementation, merits the application of novel diagnostic tools.

The ability of MALDI Imaging Mass Spectrometry (IMS) to analyze a wide range of biomolecules directly from discrete regions of tissue sections provides spatial proteomic information that can be used with histopathological findings to correctly classify diagnostically challenging samples.[23,24] MALDI IMS has demonstrated this ability in the diseased tissue of lung tumors, prostate cancer, thyroid cancer, oral squamous cell carcinoma, renal cell carcinoma, breast cancer, brain cancer, ovarian carcinomas, and melanoma.[23,25,26] Existing proteomic studies on cutaneous diseases are varied. Proteomics studies have provided insight in disease pathogenesis, diagnostics, and prognostics in other cutaneous diseases, including psoriasis, atopic dermatitis, and contact dermatitis.[27,28] Regarding malignancy, protein biomarkers such as cytokines have been utilized to monitor the immune system response to melanoma, as well as to differentiate cutaneous squamous cell carcinoma from its precursor keratinocytic skin lesions, mitigating the potential negative impact of misdiagnosis or delayed diagnosis on patient outcome.[29,30] In our previous work, we have established that proteomic profiles acquired using MALDI IMS can accurately diagnose melanocytic lesions.[22,31]

**Fig 2:**
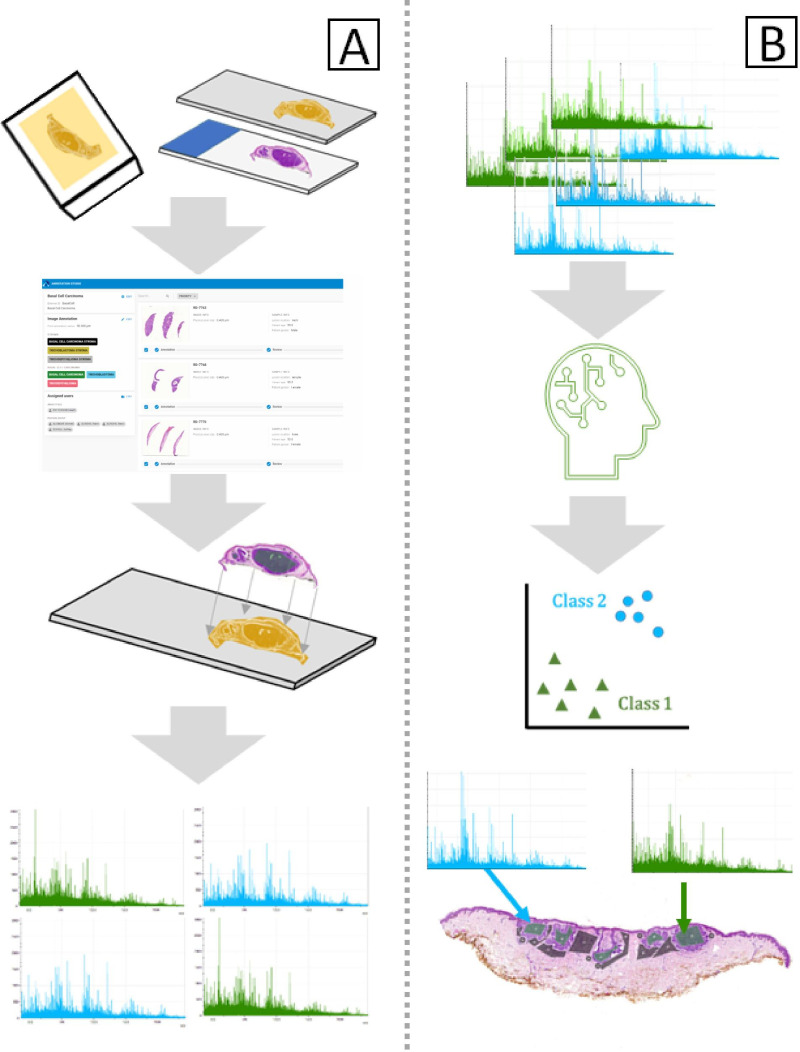
Workflow for histology-directed IMS. A) Cases are selected, and blocks are sectioned to include a slide for annotation and a slide for IMS. A pathologist annotates regions of interest registered to the section for IMS. Mass spectra are collected from the area annotated. B) Mass spectra are binned based on the annotations by pathologists and subjected to machine learning, which develops classifiers based on spectral features. The model can classify novel data and generate predictions based on spectral features.

However, no proteomics studies in differentiating between BCC and TB/TE have been published. This study aims to prove the feasibility of histology-directed MALDI IMS data to correctly differentiate BCC from TB/TE in formalin-fixed paraffin-embedded (FFPE) skin biopsies by using cases of known diagnosis to identify differences in the proteomic profile of diagnostically concordant and non-challenging cases. Fig 2 shows the workflow implemented in the study, where data are collected from serial tissue sections using pathological annotations as a guide. The resulting mass spectra were used to train a support vector machine classification model, which identifies unique spectral features indicative of each class.

## Materials and methods

### Selection of cases

The study was reviewed by Sterling Institutional Review Board (Atlanta, GA) and determined to be exempt from IRB review under the terms of the U.S. Department of Health and Human Services Policy for Protection of Human Research Subjects at 45 C.F.R. §46.104(d). FFPE tissue blocks identified with BCC and TE/TB were selected from the Pathology Associates of St. Thomas archives. Enrolled samples were selected after searching that pathology database for biopsied/excised samples with sufficient tissue before September 2021. The data collected included the age, gender, tumor location, and final diagnosis for each case.

### Histopathologic analysis

Three board-certified dermatopathologists reviewed 71 candidate specimens of BCC and TB/TE, and 69 specimens with sufficient material for testing were included. Cases were randomly designated as training set (n = 55) and validation set (n = 14). Table 1 details specific patient demographics included in the training and validation sets.

**Table 1:** Demographics for Training and Validation Sets. Numbers represent unique patients.

Mean age		Training Set (n = 55)	Validation Set (n = 14)
**BCC**		69	60
**TB/TE**		48	51
**Gender**			
**BCC**	Male	26	8
	Female	7	2
**TB/TE**			
	Male	6	1
	Female	16	3

Blocks were sectioned at 6 µm thickness. Serial sections were collected for pathological analysis and mass spectrometry analysis. Sections were mounted onto a standard glass microscope slide for hematoxylin and eosin (H&E) staining or an indium-tin-oxide (ITO)-coated glass slide (Delta Technologies, Loveland, Co) for mass spectrometry analysis. Each ITO slide contained 2–3 specimens where space allowed. Samples were randomly distributed across the slides. Digital whole slide images of the H&E-stained sections were acquired using a Huron TissueScope LE120 (Huron Digital Pathologies) using a 20✕ objective with a standard tube lens and a 1.5✕ Barlow lens for a total magnification of 30✕. Digital whole slide images were uploaded to a digital annotation software (Annotation Studio, Aspect Analytics, Genk, Belgium) for pathologists to use for remote annotation. Images were annotated using a 100 µm diameter spot tool or a free-form region of interest tool. Regions of interest were spot-filled using 100 µm spot at a uniform 150 raster. Samples were annotated using these classes: BCC epithelial elements, BCC stroma, TB/TE epithelial elements, and TB/TE stroma.

The annotation spots of 100 microns were placed to maximize the amount of tissue analyzed without overlapping data points. The analysis excluded regions of tissue where multiple epithelial/stromal elements showed significant overlap. Spots were placed on tissue areas that comprised either epithelial or stromal elements, not both.

### Mass spectrometry analysis & tissue classification

Serial sections from the annotated slides used for MALDI analysis were deparaffinized using a series of xylene and graded ethanol solutions as previously described.[11] Briefly, slides were placed in an oven at 55°C for 1 hour to soften the paraffin. The slides were deparaffinized sequentially in jars containing xylenes, fresh xylenes, 100% ethanol, fresh 100% ethanol, 95% ethanol, 70% ethanol, ultrapure water, and fresh ultrapure water. Slides were continuously agitated in each solution. Following deparaffinization, slides were subjected to heat-induced epitope retrieval (HIER). A Decloaking Chamber NxGen (Biocare Medical, Pacheco, CA) was used to warm EDTA Decloaker solution (pH 8.5) (Biocare Medical, Pacheco, CA) to 95°C. Deparaffinized slides were added to the heated buffer and heated for 40 minutes. Following heat-induced epitope retrieval, slides were buffer exchanged four times using ultrapure water to remove salts. Slides were allowed to dry upright in an incubator before they were scanned unstained at 30✕ for registration of pathological annotation. Before enzymatic digestion, 1 µ L of 1 µg/ µ L bovine serum albumin was spotted on the surface of the slide and away from tissue to serve as a digestion quality control standard.

Following HIER, fiducials were marked on the slide using a marker. The slides were scanned using the Huron TissueScope LE120 and were used to register pathologist annotations with a custom python script which uses elastix registration software to transfer the pathology annotations to the flexImaging teach image and then provides the coordinate positions of each pathology annotation in the teaching image space which ultimately guides data acquisition within the MALDI MS instrument. The generated coordinates were used on day 2 to collect data from annotated regions.

Tryptic digestion was performed using an automatic sprayer, similar to the method previously described.[26,32] Dimethylated, proteomics-grade trypsin was purchased as a lyophilized powder in 100 µg aliquots. Trypsin was reconstituted in 100 mM acetic acid to a concentration of 0.5 µg/ µ L and stored at -20°C until ready for use. Trypsin was prepared immediately before application to prevent autolysis. A 100 µ L aliquot of 0.5 µg/ µ L trypsin was combined with 513 µ L of 100 mM ammonium bicarbonate and 62 µ L of acetonitrile. The solution was vortexed and pH tested before use. Trypsin was applied using an M5 Sprayer (HTX Technologies, Chapel Hill, NC) equipped with a syringe pump (KDS Series 100 Single Syringe Pump). Eight passes of trypsin were applied to the tissue at a flow rate of 0.96 mL/hour at a velocity of 1500 mm/min in a crisscross pattern with 2 mm spacing. Nitrogen was used as a carrier gas at 10 psi. Following digestion, slides were sealed face-up in a Petri dish containing a Wyp-all paper towel wetted with 513 µ L of 100 mM ammonium bicarbonate and allowed to digest overnight at 37°C.

Following digestion, the matrix was applied to the slides. The MALDI matrix α-Cyano-4-hydroxycinnamic acid (CHCA) was prepared at 5 mg/mL concentration in 90% acetonitrile with 0.2% trifluoroacetic acid. The mixture was sonicated before spraying. Matrix was applied using an M5 Sprayer (HTX Technologies) and a Knauer P4.1S Pump. The nozzle on the M5 sprayer was heated to 72°C with a Nitrogen sheath gas at 10 psi. Matrix was applied in 6 passes at a flow rate of 0.16 mL/min, a nozzle velocity of 2600 mm/min in a crisscross pattern with 2 mm track spacing.

Mass spectra were collected from areas annotated by dermatopathologists using an ultrafleXtreme (Bruker Daltonics) MALDI-time-of-flight (TOF) MS equipped with a SmartBeam Laser (Nd:YAG, 355 nm.). For each 100 µm spot annotated, a unique mass spectrum was collected. The instrument was operated in reflector positive mode with a mass range of *m/z* 700–3500 collected. Ions under *m/z* 500 were suppressed. A total of 500 laser shots were collected from each location without random walk. The laser was set to the “medium” setting. The data was collected in typewriter model for each specimen.

Mass spectral preprocessing and statistical analysis were performed in R using in-house scripts. Mass spectral preprocessing was performed using the MALDIquant library and the following pipeline was used: TIC normalization, TopHat baseline subtraction, intensity smoothing using Savitzky-Golay algorithm, recalibration of the mass axis of all spectra to the overall mean spectrum, peak detection using signal-to-noise ratio of 3, peak binning with a tolerance of 0.2 Da, and peak filtering to remove peaks that are only present in 1% of the spectra.[33] After the peaks were extracted from the data, we created a peak matrix where columns are peaks, rows are samples, and the corresponding class labels are associated with each specimen.

The data was randomly split 80/20 into a training (n = 55) and a validation set (n = 14) at the sample level to avoid contamination of the training data with mass spectra from the validation set that would occur strictly at the spectral level. A support vector machine (SVM) classification model with a linear kernel was trained from the training set with 5-fold cross-validation with additional calculation of class probabilities. The test set was submitted to the model, and classifications were returned. For each sample group, we generated class-wise mean spectra and one-vs-all receiver operating characteristic curves to identify strongly differentially expressed peptides across classes.

## Results

**Fig 3:**
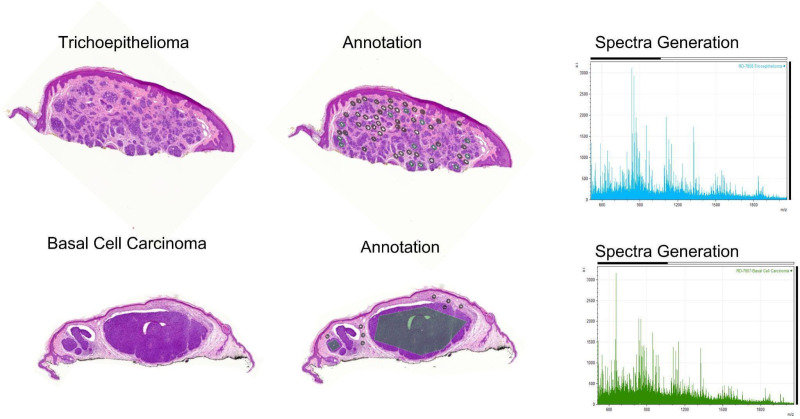
The top row demonstrates a case of Trichoepithelioma, and the lower row represents a case of Basal cell carcinoma. Note the different generated spectra from these two tumors despite the histopathologic overlap (x-axis representing *m/z* (mass-to-charge) and y-axis representing ion intensity). Mass Spectra are displayed from *m/z* 500–2100 and represent a subset of the mass range collected.

This study collected mass spectra from 43 BCC samples, resulting in 5,652 spectra from BCC nests and 2,711 spectra from the stroma. Mass spectra were also collected from 26 TB/TE samples, resulting in 1,285 spectra from TB/TE nests and 2,539 spectra from spindled stroma. These samples were divided into a validation set of 14 (10 BCC and 4 TB/TE) and a training set of 55 samples (33 BCC and 22 TB/TE). Three models were trained from MALDI data acquired from the 55 BCC and TB/TE samples from the training set. Model 1 examined BCC stroma compared to TB/TE stroma, Model 2 examined BCC tumor nests compared to TB/TE tumor nests, and Model 3 examined BCC tumor nests and stroma combined compared to TB/TE tumor nests and stroma combined. Fig 3 demonstrates two examples from the study (trichoepithelioma on top and Basal cell carcinoma in the bottom) with how the annotation was performed and how the generated spectra from each sample showed differences.

### Training results

Training (Self-recognition) is the ability of a model to classify the data from which it was trained correctly. The spectra from the 55 samples used to generate the models were tested in these instances.

Model 1 (BCC stroma vs. TB/TE stroma) self-recognition based on stroma alone analyzed a total of 3955 spectra. The model demonstrated an assay performance with a sensitivity of 91.2% (95% CI 89.7–92.5%) and specificity of 100% (95% CI 99.8% to 100%).

Model 2 (BCC tumor nests vs. TB/TE tumor nests) self-recognition based on tumor nests alone. This model included a total of 5158 spectra and demonstrated an assay performance with a sensitivity of 100% (95% CI (99.9–100%) and specificity of 50% (95% CI 47.2–52.9%).

Model 3 (BCC tumor nests/stroma vs. TB/TE tumor nests/stroma) self-recognition based on stroma and tumor nests combined. This model included a total of 9113 spectra and demonstrated an assay performance with a sensitivity of 100% (95% CI 99.9%-100%) and specificity of 86.5% (95% CI 85.3–87.6%). Table 2 depicts the self-recognition findings of MALDI IMS for each model.

**Table 2:** Training (Self-recognition) Results. These data describe spot-level classification from 55 samples. Each spot generates a unique averaged mass spectrum.

	Model 1: Stroma Self Recognition (Spot)		
	BCC	TB/TE	Assay Performance
BCC	1512	146	91.2% (CI 89.7% to 92.5%)
TB/TE	0	2297	100% (CI 99.8% to 100%)
	Model 2: Tumor Nests Self Recognition (Spot)		
	BCC	TB/TE	Assay Performance
BCC	3942	0	100% (CI 99.9% to 100%_
TB/TE	607	609	50% (CI 47.2% to 52.9%)
	Model 3: Combined Assay Self Recognition (Spot)		
	BCC	TB/TE	Assay Performance
BCC	5600	0	100% (CI 99.9% to 100%)
TB/TE	476	3037	86.5% (CI 85.3% to 87.6%)

### Validation results

Validation results are based on 14 samples that were naive to the model. The validation set provides an unbiased metric for the assay performance.

Model 1 (BCC stroma vs. TB/TE stroma) validation based on stroma alone included 1295 spectra. This model demonstrated an assay performance with a sensitivity of 46.1% (95% CI 43.0–49.1%) and specificity of 99.2% (95% CI 97.1–99.9%).

Model 2 (BCC tumor nests vs. TB/TE tumor nests) validation based on tumor nests alone included 1779 spectra. This model demonstrated an assay performance with a sensitivity of 98.9% (95% CI 98.3–99.4%) and specificity of 88.4% (95% CI 78.4–94.5%).

Model 3 (BCC tumor nests/stroma vs. TB/TE tumor nests/stroma) validation based on stroma and tumor nests combined included 3074 spectra. This model demonstrated an assay performance with a sensitivity of 90.3% (95% CI 89.1–91.3%) and specificity of 97.1% (95% CI 94.6%-98.7%). Table 3 depicts the validation findings of MALDI IMS for each model.

**Table 3:** Validation Results. These data describe spot-level classification from 14 samples that were naive to the original classifier. Each spot generates a unique averaged mass spectrum.

	Model 1: Stroma Validation (Spot)		
	BCC	TB/TE	Assay Performance
BCC	485	568	46.1% (CI 43.0% to 49.1%)
TB/TE	2	240	99.2% (CI 97.1% to 99.9%)
	Model 2: Tumor Nests Validation (Spot)		
	BCC	TB/TE	Assay Performance
BCC	1692	18	98.9% (CI 98.3% to 99.4%_
TB/TE	8	61	88.4% (CI 78.4% to 94.5%)
	Model 3: Combined Validation (Spot)		
	BCC	TB/TE	Assay Performance
BCC	2494	269	90.26% (CI 89.1% to 91.3%)
TB/TE	9	302	97.1% (CI 94.6% to 98.7%)

## Discussion

### Novel molecular analysis can augment a pathologist’s diagnosis of a disease

Traditionally, histopathological evaluation is the current standard of care for diagnosing BCC, while supplemental dermoscopy is used to improve prebiopsy accuracy and differentiation from other neoplastic and inflammatory disorders; however, pitfalls remain.[12,34] The shared appearance of tumor nests of proliferating basaloid cells with peripheral palisading and arborizing vessels with gray ovoid nests and globules seen on histology and dermoscopy, respectively, can impede a Dermatopatholgist’s ability to distinguish between BCC and its benign mimic TB/TE.[18] To clarify further, immunohistochemical staining is often utilized; however, it does not confer an optimal degree of accuracy in patient outcomes and guidance on the proper course of treatment.

MALDI IMS is a technology that combines the molecular specificity of mass spectrometry from areas of interest within tissue.[35,36] MALDI IMS has previously been used to generate protein signatures that can correctly classify malignancies of the skin.[22,23,31,37–42] MALDI IMS can also distinguish between BCC and its benign mimic, TB/TE. This novel diagnostic tool has demonstrated its potential to aid Dermatopathologists in differentiating between lesions that share similar characteristics, both clinically and histologically. In contrast to other molecular assays in dermatopathology, this method required only two sections from paraffin-embedded tissue sectioned at 6 µm thickness (one for H&E staining and the second for spectra collection) with features of interest sampled within a 100 µm area. This is amenable to assessing small tissue samples and biopsies with minimal tumor burden.

### Generation of machine learning models from MALDI IMS data

This work presents three models used to differentiate BCC and TE/TB. The models were trained from a cohort of 33 BCC and 22 TE/TB resulting in a total of 9,113 mass spectra. From tumor nests, 5158 spectra were included, with 3942 from BCC and 1216 from TE/TB. From stroma, 3955 mass spectra were included, with 1658 from BCC and 2297 from TE/TB. In general the BCC samples were larger and supported the collection of more spectra. Because of this, the study is not ideally balanced on a spectral or sample level. The model was taught with each mass spectrum representing a measurement equally in the model with no patient level consideration. Therefore, larger biopsies could be overrepresented because they contributed more spectra. This could contribute to the lower than expected self recognition of Model 2, which classified TE/TB tumor nests as BCC approximately 50% of the time. Future models should consider enrolling more TE/TB than BCC to accommodate for the smaller biopsies and increase the number of spectra from these tumor nests.

For this initial proof of concept study, a basic machine learning model using support vector machines was implemented to demonstrate the performance of MALDI IMS in this indication. Advanced machine learning models based on more modern techniques like deep neural networks or unsupervised learning could enhance classification performance.[43–46] Additionally, multimodal models can be generated that integrate not only the molecular information but also the histopathological data from the annotation.[31] These approaches require more sophisticated computational tools and are beyond the scope of this proof-of-principle exercise; however, these approaches are valuable for the final optimization of the predictive algorithm.

### MALDI IMS differentiates BCC and TE/TB

The validation samples, independent patients from those used to train the classifier, included 4 TB/TE and 10 BCC. There were 1710 spectra from the BCC and 69 spectra from TB/TE tumor nests. An additional 1053 spectra from BCC stroma and 242 spectra from TB/TE stroma were included. These samples represent a way to test the models in an unbiased way using new data.

Model 1, which considered stroma alone, proved highly reliable for identifying TB/TE with a specificity of 99.2% (240 correct spectra) but struggled to identify all cases of BCC effectively, with a sensitivity of 46.1% (485 correct spectra and 568 incorrect). Model 2 considered only tumor nests and had 98.9% sensitivity and 88.4% sensitivity, correctly classifying 1692 spectra from BCC and 61 from TB/TE. These results support that tumor nests contain distinct spectral features unique to BCC that enable more accurate prediction of the clinical phenotype than stroma alone. Stroma can be more variable and may overlap in its spectral features between different types of tumors, reducing the accuracy of differentiating between BCC and TB/TE based on stroma alone. In theory, combining tumor nests with stroma could potentially provide a more comprehensive biochemical profile, enhancing the ability of MALDI-IMS to accurately classify specimens.

Model 3 combined tumor nests and stroma into a single model. The validation set considered 3074 spectra, with 2763 from BCC and 311 from TB/TE. This model demonstrated a sensitivity of 90.3%, correctly classifying 2494 spectra as BCC, and a specificity of 97.1%, correctly classifying 302 TB/TE mass spectra. Model 3 shows a slight drop in sensitivity from model 2 but experiences an increase in specificity. This performance suggests that the combination of features may offer additional advantages in practice, such as improved robustness or generalizability. Both models 2 and 3 should continue to be evaluated using additional criteria such as ease of implementation, computational efficiency, or other practical considerations, Model 3’s combination of features might offer practical benefits beyond what is captured by sensitivity and specificity alone.

Given our previous reasoning that tumor nests are more likely to contain distinct and specific mass spectrometry signatures as opposed to stroma, we offer potential explanations for these findings. From a pathological point of view, TB/TE samples may exhibit more variability in their stromal elements on deeper sections and tend to occupy smaller geographic areas compared to tumoral nests, thus affecting the models’ performance. Regarding IMS, the models’ training and specificity may also play a role in this discrepancy. That is, if trained or validated predominantly on BCC samples, then this could make it less adept at recognizing the variations found in TB/TE samples.

This approach to the collection of IMS data utilized a serial section from the one annotated for molecular analysis. There is a potential for changes in tissue histology for small features in serial sections. An approach to circumvent this is to image the entirety of the slide and utilize unsupervised machine learning to define mass spectral features that indicate tissue features. In these approaches, the MALDI matrix can be removed, and the section can be stained post-analysis. [47–49] However, there can be damage to the tissue in the process, making post-analysis pathological evaluation of the same section difficult. Additionally, the IMS may have had different sensitivities for different tissue components due to differences in tissue density or ionization efficiency.

### Patient level scoring

This study only included concordant cases that were not diagnostically ambiguous. Thus, every spectrum was scored as a unique test in the data presented above. However, within the 14 test samples, it would be advantageous to have a summary of all the spectra within the section to assist in the diagnosis of the case. For example, consider a biopsy with 10 spots. If 2/10 were classified as BCC, the data could hinder diagnosis rather than ease the decision. There is a broad range of mass spectra per biopsy in the validation set, ranging from 10 to 1643. Each patient can be considered individually with the number of spots classified and the total percent correct. Table 4 represents these data per sample as generated in model 3. The percent correct ranges from 48 to 100% of spectra classified correctly. Therefore, setting thresholds for a positive patient must be considered. Calibration of the patient-level scoring requires a significant expansion of the training set to determine a minimal number of spots required for the assay to make a prediction and set thresholds for scoring accurately. Additionally, the inclusion of other metadata, including additional machine learning on histological images has been shown to improve the performance of MALDI-based classifiers.[50–52] Furthermore, in the context of patient care when appropriate consent is obtained, the combination of molecular, imaging, and clinical data can be used together to strengthen classification models.[50,53,54] A similar approach could yield an improvement in patient-level scoring.

**Table 4:** Summary of patient-level predictions of Model 3 in the Validation set. Spectra that were classified correctly are displayed in black, where spectra classified incorrectly are shown in red. There is a large variance in the number of spectra per biopsy and the overall percent correct.

Patient no.	Diagnosis	N Spots	BCC	TB/TE	% Correct
**1**	BCC	10	9	1	90
**2**	BCC	14	14	0	100
**3**	BCC	44	42	2	95
**4**	BCC	38	38	0	100
**5**	BCC	67	58	9	87
**6**	BCC	1643	1629	14	99
**7**	BCC	129	111	18	86
**8**	BCC	207	99	108	48
**9**	BCC	444	380	64	86
**10**	BCC	167	114	53	68
**11**	TB/TE	185	3	182	98
**12**	TB/TE	50	1	49	98
**13**	TB/TE	51	5	46	90
**14**	TB/TE	25	0	25	100

## Conclusion

MALDI IMS is a proven analytical tool in the field of dermatopathology. In this proof of principle study, the SVM model trained on the peptide signatures acquired with MALDI IMS differentiated BCC from TB/TE using a combination of stroma and tumor nests with a sensitivity of 90.3% and specificity of 97.1% in a validation set of 14 samples. A model using only tumor nests had a sensitivity of 98.9% and a specificity of 88.4%. While these findings are not yet validated for clinical use, they indicate a significant difference in protein contents aiding in the differentiation between BCC and TB/TE. Thus, this merits further research to support implementing MALDI IMS as an ancillary diagnostic tool for adequately and efficiently identifying the most common cutaneous malignancy in the United States.

The limitations of this study included a small validation set (n = 14), which included easily identifiable cases obtained from a single institution. In general, TB/TE samples were smaller and fewer spots were placed than the BCC biopsies. A larger cohort, including a subset of histologically challenging cases from multiple institutions with adequate clinical follow-up to confirm diagnostic accuracy, will be required to fully validate the efficacy of MALDI IMS in differentiating between BCC and TB/TE and thus supporting its integration into clinical practice.
